# Optimal Strategies for Reducing Number of People in the Social Security System

**DOI:** 10.3390/ijerph17041305

**Published:** 2020-02-18

**Authors:** Paul Yip, Mengni Chen, Bing Kwan So, Kwok Fai Lam, Kam Pui Wat

**Affiliations:** 1 Department of Social Work and Social Administration, The University of Hong Kong, Hong Kong SAR, China; 2 Centre for Demographic Research, Catholic University of Louvain, 1348 Louvain-la-Neuve, Belgium; hkfancycmn@gmail.com; 3 Mathematics Department, Jilin University, Changchun 130012, China; bkso@graduate.hku.hk; 4 Department of Statistics and Actuarial Science, The University of Hong Kong, Hong Kong SAR, China; hrntlkf@hku.hk (K.F.L.); watkp@hku.hk (K.P.W.)

**Keywords:** poverty rate, social security system, youth mobility, effectiveness

## Abstract

Providing social security to the population in need has become a major expenditure for many governments. Reducing the number of dependents in the social security system and maintaining a dynamic economically active population is a high priority concern for policymakers. A good understanding of the dynamics of the social security system—specifically, who enters and who exits the system—would be helpful for formulating effective interventions. Here, we made use of the data of Hong Kong’s Comprehensive Social Security Assistance (CSSA), which is currently a basic welfare scheme in Hong Kong that provides supplementary payments to households that cannot support themselves financially. We proposed a stochastic model to examine the in- and out- movement in the CSSA scheme and conducted elasticity analyses. The elasticity analyses allowed us to identify the potential target groups of people that would lead to the largest reduction in the number of the CSSA recipients in the system. This analytical method can also reveal whether policies would be more effective in preventing people from entering the CSSA system or helping them leave the CSSA scheme. Our analyses suggest that targeting those aged 30–49 with children would have the largest impact. Additionally, we found that policies that aim to prevent this group from entering the CSSA system would be more effective in reducing the number of CSSA recipients compared with policies that aim to help them exit. In contrast, for the younger age group of 10–29, policies that help them leave CSSA would be more effective than policies that prevent them from entering CSSA. Providing employment for those unemployed in this younger group would be more effective. The results indicate that by tailoring measures to specific subgroups, the overall number of CSSA recipients would be reduced, thereby improving the efficiency of Hong Kong’s social security system, which has accounted for more than 16.5% of Hong Kong government expenditure in 2018, amounting to more than HKD 92 billion.

## 1. Introduction

“Leave no one behind” is the overarching principle of the United Nations’ 2030 Sustainable Development Goals [1]. Ending poverty everywhere, in all its forms, is the first of the Sustainable Development Goals (SDG). In signing the SDG Agenda 2030, governments around the world have committed to achieving this goal over the coming years. Hong Kong is one of the richest cities in the world with an impressive GDP of USD 46,000, but with a large Gini coefficient of 0.537, and thus the issue of poverty deserves special attention. The poverty line in Hong Kong is set at 50% of the median monthly household income before taxation and the government’s intervention (e.g., allowances for the elderly and low-income families). According to the Hong Kong Poverty Situation Report in 2017, there were 1.377 million persons living below the poverty line in Hong Kong, accounting for 20.1% of the total population [2]. After taking account of the government recurrent cash benefits, the size of the poor population reduced to 1.009 million, accounting for 14.7% of the population. Researchers have argued that poverty can be alleviated by social security if it can effectively redistribute resources towards the poor [3,4], and local statistics do prove that social security can make a significant difference to Hong Kong’s poverty situation [2].

Providing social security to the poor population is a major public expense. For 2018–2019, it is estimated that the Hong Kong government’s total social welfare expenditure would rise to HKD 92.2 million, occupying 16.5% of the total expenditure—the second largest cost after education [5]. A large expenditure on social security has always been a concern to governments, especially in times of unfavorable economic conditions and rapid population aging. The Basic Law of Hong Kong Special Administrative Region (Hong Kong SAR) makes it clear that the development and expansion of social welfare is only possible when the economic conditions allow, through the following, “On the basis of the previous social welfare system, the Government of Hong Kong SAR shall, on its own, formulate policies on the development and improvement of this system in the light of economic conditions and social needs” [6]. The present Chief Executive of Hong Kong, Carrie Lam, also highlighted, “the requirements stipulated in Basic Law of keeping expenditure within the limits of revenues and avoiding fiscal deficits as far as possible” [7]. This fiscal conservatism is coupled with emphasis on economic growth, making the government inclined to promote the ideology of self-reliance in delivering social policies. On this basis, reducing the number of dependents in the social security system and maintaining a sizable economically active population has become a goal for policymakers in Hong Kong.

Although the Hong Kong government has made some efforts, in one way or another, to reduce the financial burden of social security, there have been many controversies over their approaches [8,9,10]. Some critics have questioned the effectiveness of the social security system in alleviating poverty, and there has been very limited knowledge about whom to target, and in which form, so that the social security system in Hong Kong cannot only reduce poverty but also keep within the government budget. This study tries to fill the gap by evaluating Hong Kong’s Comprehensive Social Security Assistance (CSSA) scheme—the most important assistance program for those in need. Specifically, we examined the dynamics (i.e., inflow and outflow) of the CSSA system on the basis of a stochastic model, utilizing a Markov chain process. We performed elasticity analyses to (1) identify the potential target groups of people for interventions that would lead to the largest reduction in the number of the CSSA recipients and (2) reveal whether it would be more effective to pursue policies that prevent people from entering the CSSA system or policies that help people leave CSSA. The findings of our analysis will provide insights for further improving the effectiveness of the social security system in Hong Kong.

## 2. Background

Until 1997, Hong Kong was a British colony for more than 150 years (since 1842). The social security system in Hong Kong was shaped by the laissez-faire philosophy under the previous colonial regime. The concept of self-reliance via employment is deeply rooted in the psyche of the Hong Kong population, and the so-called Hong Kong “Lion Rock Spirit” captures an ethic of hard working and mutually helping one another in the community. Only the very needy and vulnerable families would receive support by the government. Thus, Hong Kong’s welfare system was perceived as a typical example of the residual model of welfare, which views the government welfare provision only as a last resort. The laissez-faire philosophy and residual welfare model were also compatible with Chinese Confucian values, which emphasize filial piety, respect for older people, love for one’s family, self-restraint, shouldering collective responsibility, mutual help, and so on. Chiu and Wong (2005) [11] have argued that Confucian ideology has been used by the Hong Kong government as “a means to contain social welfare costs”, as well as to justify the residual welfare model.

On July 1st of 1997, the sovereignty over Hong Kong was returned back to Mainland China and the city has become a Special Administrative Region of the People’s Republic of China (HKSAR). Under the principle of “one country, two systems”, Hong Kong has enjoyed a high degree of autonomy and is responsible for its domestic affairs including tax system. The HKSAR government does not have to contribute any revenue towards the Central Government. Since 1997, the social policies in Hong Kong have been not only shaped by the laissez-faire philosophy that were “inherited” from the colonial regime, but also constrained by the Basic Law that was issued after the handover to China [10]. According to the Basic Law, the Hong Kong government can only increase its public expenditure on social welfare within the limits of the budget surplus [12]. Thus, social policies have become sensitive to the government budget. Since 1997, the government experienced 6 years of budget deficit, from 1998–1999 to 2004–2005, which was related to the 1997 Asian financial crisis and the 2003 Severe Acute Respiratory Syndrome (SARS) epidemic. During this period, the low-income group suffered most from the two economic shocks. The rising social welfare needs of low-income families greatly increased government spending, and consequently new social policies to expand and diversify the social welfare system had to be suspended [10].

**Figure 1:**
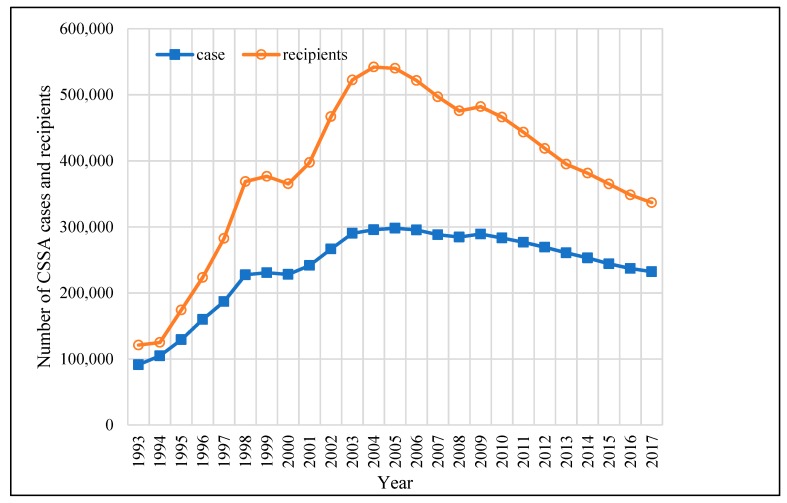
The numbers of Comprehensive Social Security Assistance (CSSA) cases and recipients over the period 1993–2017. Source: Statistics on Comprehensive Social Security Assistance Scheme 1993–2017 provided by the Hong Kong Census and Statistics Department.

The Comprehensive Social Security Assistance (CSSA) is the most important welfare program in Hong Kong. Previously, this social security was called “public assistance”, which was introduced in 1971 and modified from the British National Assistance Act. Public assistance aimed to help the aged and the sick to maintain a basic living standard with a minimal allowance. It was in 1993 that public assistance was renamed CSSA. The applications of CSSA have to go through income and assets means tests. In the first year (i.e., 1993), the number of CSSA cases was 91,362 and the number of CSSA recipients was 121,060. There can be more than one recipient in one case, as the household is treated as a case unit and all the members within the household can be recipients. Figure 1 shows the trends of CSSA cases and recipients over the period of 1993–2017. The number of cases rapidly increased and reached a peak in the year 2005 (of 298,011); since then, it has steadily declined to 232,134 in 2017. The number of recipients had a similar trend, with a dramatic increase in the years of 1997, 1998, 2002, and 2003. In 1997 and 1998, when the Asian financial crisis struck Hong Kong, the number of recipients had increased by 59,239 and 86,000, respectively.

**Figure 2:**
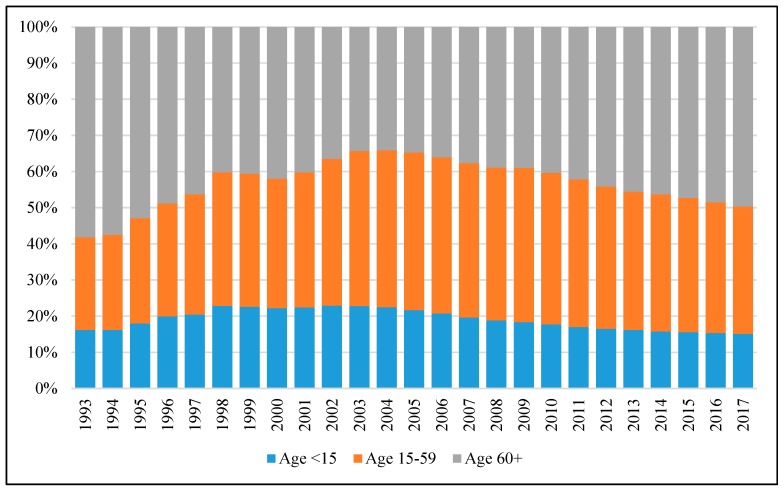
The age composition of CSSA recipients over the period 1993–2017. Source: Statistics on Comprehensive Social Security Assistance Scheme 1993–2017 provided by the Hong Kong Census and Statistics Department.

Figure 2 shows the age composition of the recipients. As shown, in 1993, those aged 60 and above accounted for almost 60% of recipients, whereas those aged 15–59 and under 15 occupied about 25% and 15%, respectively. The proportion of recipients aged 0–59 initially rose continuously, reaching about 66% in 2004; since then, it has started to decline and the proportion was about 50% in 2017. As the population continues to age rapidly due to low fertility and long life expectancy, the proportion of recipients aged 60 and above is expected to increase.

**Figure 3:**
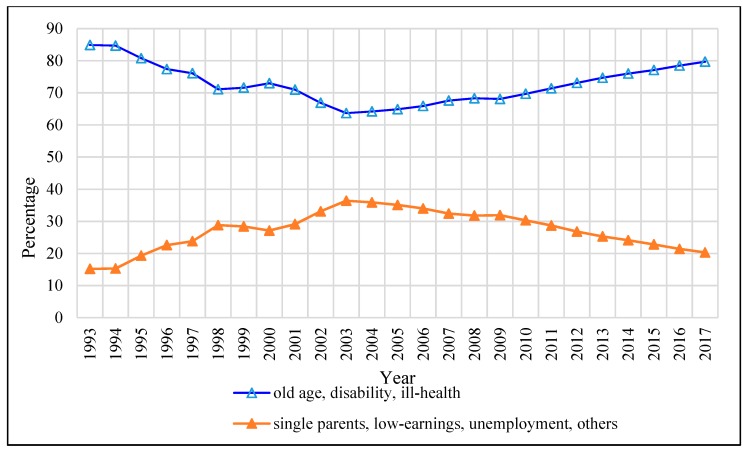
The distribution of CSSA by category over the period 1993–2017. Source: Statistics on Comprehensive Social Security Assistance Scheme 1993–2017 provided by the Hong Kong Census and Statistics Department.

Figure 3 shows the distribution of CSSA cases by category. The proportion of CSSA cases under old age, permanent disability, and ill-health declined from 84.9% to 63.7% in the period of 1993–2004, and rose gradually after 2004, reaching 79.7% in 2017. The proportion of cases involving single parents, low-earners, the unemployed, and others, increased from 15.2% to 36.4% over the period of 1993–2003—with a particularly significant increase of 5% in 1997 due to the Asian financial crisis, and then started to decline during 2004–2017. In 2017, about 20.3% of the CSSA cases fell into these categories.

**Figure 4:**
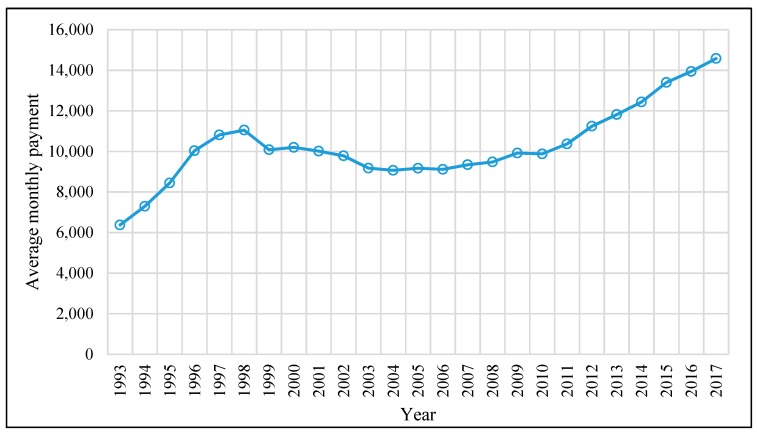
The average monthly CSSA payments to a household with four eligible members over the period of 1993–2017. Source: Statistics on Comprehensive Social Security Assistance Scheme 1993–2017 provided by the Hong Kong Census and Statistics Department.

Figure 4 shows the average monthly CSSA payments to a household with four eligible members. The payments increased from HKD 6374 in 1993 to HKD 11,050 in 1998. When the government faced a budget deficit during 1998–2004, the payment was gradually reduced to HKD 9068 in 2004. It was not until 2012 that the payment reversed to the level in 1998. Since then, the payment has increased continuously to HKD 14,579. In 2017/2018, the government expenditure on CSSA was HKD 20.6 billion—an increase of more than HKD 10 billion from HKD 9.4 billion in 1997/1998. It was the largest rate of increase among all expenditures.

Such a large share of spending on CSSA and its rate of increase have become a concern for the Hong Kong government, especially in the years of the Asian financial crisis and SARS epidemic, when the budget turned to a deficit. The government did take steps to control the expenditure on CSSA and balance their budget by reducing the value of CSSA payments and the number of recipients. Theoretically, reducing the number of recipients can be achieved either by preventing people from entering the CSSA system or helping them leave the system. The “entering approach” often influences a relatively larger number of the population at risk of poverty, preventing “potential candidates” from falling into CSSA with more universal measures. The universal measures often include minimum wage, universal health insurance, subsidized education, public housing, and so on, from which both CSSA recipients and non-CSSA people can benefit [13]. In particular, the universal Cash Handout Scheme in Hong Kong is a very typical example. The Hong Kong government introduced Scheme HKD 6000 and Scheme HKD 4000 in 2011 and 2019, respectively. Under these schemes, any Hong Kong permanent resident aged 18 and above was eligible to get a lump-sum payment of HKD 6000 and HKD 4000 in 2011 and 2019, respectively. In contrast, the “leaving approach” is more likely to affect a relative smaller group of people that are unemployed, in low-paid work, or single parents, helping them get out of the system with more focused measures. The focused measures include single parent allowance, transportation subsidy for low-income families, job training programs for the unemployed, and so on [13].

The Hong Kong government seemed to have adopted an “entering approach” in the 2000s, but the specific means of preventing people from entering was controversial. Before 2004, Hong Kong residents could apply for CSSA if they had lived in Hong Kong for at least 1 year. In 2004, the requirement for residence was then raised up to 7 years, effective from 2004 until 2013. This revision was mainly driven by concerns about the budget shortfall [14]. Scholars also think that the government pursued the “entering approach” by blaming the poor for their laziness using the media, so that they were discouraged to apply for CSSA [8,9]. This created a psychological hurdle for those physically able adults and made them turn away from CSSA voluntarily. Wong (2000) [9] argued that the image of “laziness” and “dependency” constructed (whether intentionally or unintentionally) for the CSSA recipients actually reflected “a failure rather than a success in fighting against poverty”. The CSSA is meant to provide timely help for those needy families such that they still can receive the necessary financial support while looking for employment. It does not mean to create some dependence on the system, and those seeking help should not be stigmatized. Furthermore, it is always the children from CSSA families who would need more support to break up intergenerational poverty.

Meanwhile, it should be emphasized that “welfare dependency” is a very common problem that many social security systems would face or try to avoid [15,16,17]. When the incentives to take and sustain jobs are not strong enough, people would continue claiming benefits from the social security system as they are often more secure and attractive than employment; thus, it would create a culture of “dependency”, “laziness”, or “worklessness”, leading to “excessive and ineffective public expenditure”, as well as “persistent and prevalent poverty” [17]. To reduce such “welfare dependency”, an effective “leaving approach” may help to incentivize CSSA recipients to return to and survive in the labor market. The Poverty Commission in Hong Kong has advocated social enterprises to play a role in poverty alleviation, particularly helping welfare dependents become self-sufficient, as social enterprises would provide more opportunities to the disadvantaged, as well as improve their skills and employability [18].

It is important to consider whether the “entering approach” is indeed more effective than the “leaving approach” in reducing the number of CSSA recipients in Hong Kong. Very few studies have provided evidence for the “entering approach”. Furthermore, without reducing the CSSA payment and restricting the eligibility, is there any better way to control the expenditure on CSSA? Which group of people and in what approach should the government intervene so that the number of recipients can be effectively reduced? This paper tries to answer these mostly unexplored questions. Specifically, by modelling the dynamics of the CSSA system in a Markov chain process and performing an elasticity analysis, we aimed to identify the potential target group and the group-specific approaches. The findings can shed some light on how to enhance and optimize the social security system in Hong Kong.

## 3. Data and Method

The data required for the analysis included (1) the age-specific number of people who entered the CSSA system during 2014–2015, (2) the age-specific number of people who left the system during 2014–2015, (3) the age-specific population in 2014, and (4) the number of births by mothers in terms of age group in 2014. On the basis of these data, we estimated the age-specific transition probabilities; that is, the probabilities of leaving CSSA and the probability of entering CSSA. All of the data are from the Hong Kong Census and Statistics Department (HKC&SD).

We modelled the dynamic of people moving in and out of the CSSA system using a stochastic process (Markov chain process). This method has been increasingly used in labor economics and demographic research, especially in evaluating social policies [19,20,21,22,23]. We first present a simplified model. Suppose in a certain year, the probability of entering the system is $p_{t}$ and the probability of leaving the system is $q_{t}$. Let $x_{t}$ be the proportion of CSSA recipients in the total population at time t. Therefore, we can simply formulate the inflow and outflow of CSSA recipients in Equation (1).

(1) $$\left(\begin{array}{c} x_{t+1}\\ 1-x_{t+1} \end{array}\right)=A\left(\begin{array}{c} x_{t}\\ 1-x_{t} \end{array}\right).$$

where *A* is a 2 × 2 matrix,

$$A=\left(\begin{array}{c} 1-q_{t}\\ q_{t} \end{array}\begin{array}{c} p_{t}\\ 1-p_{t} \end{array}\right)$$

The proportion of CSSA recipients in the total population, $x_{t+1}$, can be reduced either through reducing the probability of entering CSSA, $p_{t}$, or through increasing the probability of leaving CSSA, $q_{t}$. To investigate whether the former or the latter approach is more effective in reducing the number of CSSA recipients is of great importance to policymakers.

In order to answer this question, we introduced the concept of elasticity. Elasticity is originally a concept in economics. In economics, price elasticity, for instance, measures the impact on the demand of a product when its price changes, that is, percentage change in the quantity demanded of one product in response to percentage change in the price. Very often, it is precisely interpreted as the percentage change of demand per 1% change in the price [24]. The concept of elasticity has recently been adopted by some scholars to evaluate the targeting of policies, for example the targeting of family polices in Singapore, Taiwan, and Australia [21,22,23]. Analogously, here we define the elasticity as the ratio of percentage change of $x_{t+1}$ to the percentage change of any of the parameters ($p_{t}$ or $q_{t}$). The larger the elasticity, the more influential the parameter to $x_{t+1}$. The elasticity with respect to $p_{t}$ and $q_{t}$ can be formulated as follows:

(2) $$\delta_{p_{t}}=\frac{\Delta x_{t+1}/x_{t+1}}{\Delta p_{t}/p_{t}}=\frac{\Delta x_{t+1}}{\Delta p_{t}}\cdot \frac{p_{t}}{x_{t+1}}$$

(3) $$\delta_{q_{t}}=\frac{\Delta x_{t+1}/x_{t+1}}{\Delta q_{t}/q_{t}}=\frac{\Delta x_{t+1}}{\Delta q_{t}}\cdot \frac{q_{t}}{x_{t+1}}$$

By comparing the absolute values of $\delta_{p_{t}}$ and $\delta_{q_{t}}$, we will know whether the “entering” or “leaving” approach is more effective in reducing the number of CSSA recipients in a certain year.

In reality, the probability of leaving or entering the CSSA system often varies across different age and socioeconomic groups. As the data of CSSA recipients by socioeconomic status is not available, we cannot include the socioeconomic groups in our analysis. Thus, we further elaborated our model by considering age to better reflect the real dynamics of the CSSA system in Hong Kong. Let $x_{t}^{\left(i\right)}$ and $y_{t}^{\left(i\right)}$ be the proportions of the CSSA recipients and non-CSSA people in the population at age i and time t, respectively. We note that $x_{t}^{\left(i\right)}+y_{t}^{\left(i\right)}=1$. The distribution of $x_{t+1}^{\left(i+1\right)}$ and $y_{t+1}^{\left(i+1\right)}$ at time t+1 can be modelled in Equation (4)—a modified version from Equation (1).

(4) $$\left(\begin{array}{c} x_{t+1}^{\left(i+1\right)}\\ y_{t+1}^{\left(i+1\right)} \end{array}\right)=A_{i}\left(\begin{array}{c} x_{t}^{\left(i\right)}\\ y_{t}^{\left(i\right)} \end{array}\right)$$

where $A_{t}^{\left(i\right)}$ is a 2 × 2 matrix

$$A_{t}^{\left(i\right)}=\left(\begin{array}{c} 1-q_{t}^{\left(i\right)}\\ p_{t}^{\left(i\right)} \end{array}\begin{array}{c} q_{t}^{\left(i\right)}\\ 1-p_{t}^{\left(i\right)} \end{array}\right)$$

In this study, we restricted our attention to those aged below 60, because those CSSA recipients aged 60 and above have a high chance of leaving the CSSA system due to death. The financial situation of older adults, and particularly those over the retirement age of 65, would also typically show little change due to the relatively low employability of older adults. On the basis of the data from HKC&SD, we estimated the transition parameters $p_{t}^{\left(i\right)}$ and $q_{t}^{\left(i\right)}$ for 10 year age-specific groups (covering the ages of 0–59).

Within the age group, those aged 0 deserve special attention. According to the rules under the CSSA system, the newborns of a CSSA recipient will become CSSA recipients directly. On the basis of the assumptions that the birth rates of the CSSA recipients and non-CSSA people are the same, and that the births are from women aged 15–49, $x_{t}^{\left(0\right)}$ and $y_{t}^{\left(0\right)}$ can be formulated by Equation (5).

(5) $$\left(\begin{array}{c} x_{t+1}^{\left(0\right)}\\ y_{t+1}^{\left(0\right)} \end{array}\right)=b_{t}^{\left(1\right)}\left(\begin{array}{c} x_{t}^{\left(1\right)}\\ y_{t}^{\left(1\right)} \end{array}\right)+b_{t}^{\left(2\right)}\left(\begin{array}{c} x_{t}^{\left(2\right)}\\ y_{t}^{\left(2\right)} \end{array}\right)+\cdots +b_{t}^{\left(j\right)}\left(\begin{array}{c} x_{t}^{\left(j\right)}\\ y_{t}^{\left(j\right)} \end{array}\right)+\ldots +b_{t}^{\left(59\right)}\left(\begin{array}{c} x_{t}^{\left(59\right)}\\ y_{t}^{\left(59\right)} \end{array}\right)$$

where $b_{t}^{\left(1\right)},\ldots ,b_{t}^{\left(59\right)}$ are the corresponding age-specific birth rates at time t. Certainly, $b_{t}^{\left(1\right)},\ldots ,b_{t}^{\left(14\right)}$ and $b_{t}^{\left(50\right)},\ldots ,b_{t}^{\left(59\right)}$ are equal to zero. The age-specific birth rate is the ratio of the number of births born to women aged i to the population aged i. Therefore, from Equations (4) and (5), we can see that $x_{t+1}^{\left(i+1\right)}$ and $y_{t+1}^{\left(i+1\right)}$ are functions of $p_{t}^{\left(i\right)}$, $q_{t}^{\left(i\right)}$, and $b_{t}^{\left(i\right)}$ (where i=0,…,59).

The goal of our analysis was to investigate which group should be targeted and in which approach of “leaving” or “entering”, so that the overall proportion of CSSA recipients can have the largest reduction. Let *X* denote the overall proportion of the CSSA recipients in the population aged under 60, which can be calculated from the following formula:

(6) $$X_{t+1}\overset{59}{\underset{i=0}{\sum }}x_{t+1}^{\left(i\right)}\cdot w_{t}^{\left(i\right)}$$

where $w_{t}^{\left(i\right)}$ refers to the proportion of the age group i in the total population aged under 60 at time t, and $\sum_{i=0}^{59}w_{t}^{\left(i\right)}=1$. Here, $w_{t}^{\left(i\right)}$ was estimated on the basis of the age-specific population in 2014 from HKC&SD. To achieve the goal, we estimated the elasticity with respect to $p_{t}^{\left(i\right)}$ and $q_{t}^{\left(i\right)}$ from the following formulae, which were modified from Equations (2) and (3):

(7) $$\delta_{p_{t}^{\left(i\right)}}=\frac{\Delta X_{t+1}/X_{t+1}}{\Delta p_{t}^{\left(i\right)}/p_{t}^{\left(i\right)}}=\frac{\Delta X_{t+1}}{\Delta p_{t}^{\left(i\right)}}\cdot \frac{p_{t}^{\left(i\right)}}{X_{t+1}}$$

(8) $$\delta_{q_{t}^{\left(i\right)}}=\frac{\Delta X_{t+1}/X_{t+1}}{\Delta q_{t}^{\left(i\right)}/q_{t}^{\left(i\right)}}=\frac{\Delta X_{t+1}}{\Delta q_{t}^{\left(i\right)}}\cdot \frac{q_{t}^{\left(i\right)}}{X_{t+1}}$$

where $\frac{\Delta X_{t+1}}{\Delta p_{t}^{\left(i\right)}}$ and $\frac{\Delta X_{t+1}}{\Delta q_{t}^{\left(i\right)}}$ are the rates of change, which reflect the impact on $X_{t+1}$ given a change of value in $p_{t}^{\left(i\right)}$ and $q_{t}^{\left(i\right)}$, respectively. Different from the rates of change, the elasticities reflect the percentage impact on $X_{t+1}$ given the percentage change of $p_{t}^{\left(i\right)}$ and $q_{t}^{\left(i\right)}$. We preferred the elasticity rather than the rate of change, because the former takes into consideration the easiness or feasibility of changing $p_{t}^{\left(i\right)}$ and $q_{t}^{\left(i\right)}$. Comparison of $\delta_{p_{t}^{\left(i\right)}}$ and $\delta_{q_{t}^{\left(i\right)}}$ would inform us of the potential target groups and the best approach for effective intervention.

Moreover, to better understand the moving-in and -out of the CSSA system, we calculated the probability of a CSSA recipient aged i to first exit the system at or before the age of 60, as well as his/her mean time of staying in the system before the first exit. Equations (9) and (10) were used to estimate the probability and the mean time.

(9) $$\mathrm{Pr}\left(\mathrm{leaving}\;\mathrm{at}\;\mathrm{age}\;i\right)=1-\left(1-q_{t}^{\left(i\right)}\right)\left(1-q_{t}^{\left(i+1\right)}\right)\cdots \left(1-q_{t}^{\left(59\right)}\right)$$

(10) $$\mathrm{Mean}\;\mathrm{time}=1\times q_{t}^{\left(i\right)}+2\times q_{t}^{\left(i+1\right)}\times \left(1-q_{t}^{\left(i\right)}\right)+\cdots +\left(60-i\right)\times q_{t}^{\left(59\right)}\times \left(1-q_{t}^{\left(58\right)}\right)\;\cdots \;\left(1-q_{t}^{\left(i\right)}\right)$$

## 4. Results

The estimated age-specific probabilities of entering and leaving the CSSA system are shown in Table 1. The probability of leaving was found to be much higher than the probability of entering across all the ages. The 20–29 age group has the highest probability of leaving and the lowest probability of entering the CSSA system. Except for the two youngest age groups, the probability of leaving CSSA declines with the increase of age, indicating the increasing difficulty of raising their income above the level of the threshold. On one hand, this declining trend may be due to the fact that the family size will increase with people’s age, and thus it would become more difficult to move all the family members out of the CSSA system. On the other hand, previous research shows that in Hong Kong, as men and women get older, particularly after age 30, upward mobility of earnings declines while downward mobility of earnings increases [25]. Workers in the lowest income quintile are more likely to be trapped at the bottom, experiencing no earning mobility [25,26]. Moreover, the declining probability of leaving for the two youngest age groups (i.e., ages of 0–9 and 10–19) is very much related to the parents’ probability of leaving. Parents of children aged 10-19 are more likely to be older, in the 40s and 50s, and that is probably why the leaving probability of the 10–19 age group is lower and very close to that of the 40–49 and 50–59 age groups.

Regarding the probability of entering CSSA, the 0–9 and 10–19 age groups were found to be at the highest level—much higher than the other age groups. This was because these two groups are too young to participate in the labor market and their economic situation often depends on their parents. Once their parents’ incomes fall below the threshold level of CSSA, they become CSSA recipients directly. Hence, these young people are indirectly entering the CSSA system. Thus, reducing their probability of entering should not directly aim at these young dependents but at improving incomes of their parents who are often in the age range of 30–49. Maintaining the economic environment by keeping the unemployment rate low is important for middle-aged workers.

Table 2 shows the probability of a CSSA recipient first exiting the system before age 60 and the mean duration of staying in the system. It is encouraging to see that the probabilities of first exit among those aged under 30 are higher than 0.99, implying that almost all of them will leave the CSSA system before age 60. There was a significant decline in the probability of first exit after age 30. This is very consistent with the age pattern shown in Table 1. The probability dropped to 0.73 for those aged 50 and 0.48 for those aged 55. This is because there is much less time for them to leave the CSSA before the cutoff age of 60. Regarding the mean time of staying in the system, children under age 15 were found to have the longest duration. The mean time was found to decrease to about 4.8 years for those in their 20s and then starts to rise again in the age range of 30–40. Those in their 50s have a much shorter duration of staying before they first leave the system.

**Figure 5:**
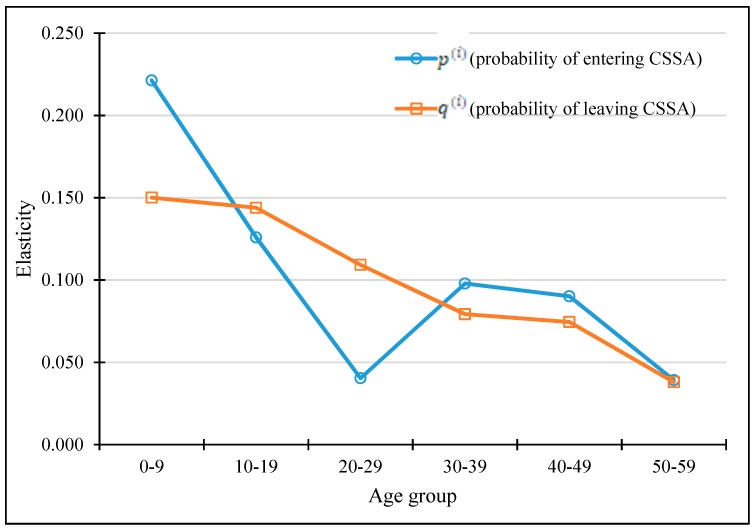
The absolute values of elasticities of the overall CSSA proportion.

On the basis of the age-specific probability of entering and leaving CSSA, we performed the elasticity analysis. The results (in absolute values) are shown in Table 3 and visualized in Figure 5. All rates of change with respect to the probabilities of entering $\left(\frac{\Delta X_{t+1}}{\Delta p_{t}^{\left(i\right)}}\right)$ were larger than the rates of change with respect to the probabilities of leaving $\left(\frac{\Delta X_{t+1}}{\Delta q_{t}^{\left(i\right)}}\right)$. For example, the rate of change with respect to $p_{20-29}$ $\left(\frac{\Delta X_{t+1}}{\Delta p_{t}^{\left(20-29\right)}}\right)$ was 0.604 and the rate of change with respect to $q_{t}^{\left(20-29\right)}$ $\left(\frac{\Delta X_{t+1}}{\Delta q_{t}^{\left(20-29\right)}}\right)$ was 0.012. If $p_{t}^{\left(20-29\right)}$ decreased by 0.1, the overall proportion of CSSA recipients would decrease by 0.0604 (= 0.604 × 0.1); if $q_{t}^{\left(20-29\right)}$ increased by 0.1, the overall proportion of CSSA recipients would decrease by 0.0012 (= 0.012 × 0.1). From these results, it seems that preventing people aged 20–29 from entering the CSSA system has a larger impact; that is, the “entering” approach would be more effective in reducing the number of CSSA recipients in 2014. However, it should be noted that it is impossible and unrealistic to achieve a decrease of 0.1 in $p_{t}^{\left(20-29\right)}$, because $p_{t}^{\left(20-29\right)}$ is only 0.0015 and a decrease of 0.1 will make it negative.

Therefore, compared to the rates of change, the elasticity that takes into account the feasibility of changing the parameter is preferable. As shown in Table 3, the elasticity to the probability of leaving in the 20–29 age group $\left(\delta_{q_{t}^{\left(20-29\right)}}\right)$ was 0.109, meaning that given 1% increase in the probability of leaving (i.e., an increase of 1% × 0.2103), the proportion of CSSA recipients would reduce by 0.109%. The elasticity to the probability of entering the group $\left(\delta_{p_{t}^{\left(20-29\right)}}\right)$× was 0.04, meaning that given a 1% decrease in the probability of entering (i.e., a decrease of 1% × 0.0015), the proportion of CSSA recipients would reduce by 0.04%. Thus, according to the elasticities, increasing the probability of leaving would have a greater impact, indicating that the “leaving approach” rather than the “entering approach” is more effective for the age group 20–29. As shown in Figure 5, the elasticities of the leaving probabilities decline with age, implying that the “leaving approach” is more effective in younger age groups than in older groups, as the employability of young people is much higher than their older counterparts and earning a salary through employment is the most effective way to leave the CSSA system. The elasticities of entering probabilities also showed a decreasing trend over ages, although the 20–29 and 50–59 age groups had the lowest elasticities. This means that the “entering approach” for these two groups is relatively less effective than in other age groups. The age pattern of the elasticities showed that targeting younger age groups would make a larger difference than targeting older groups. Therefore, intuitively, it may seem most effective to target the 0–9 age group. However, instead of focusing on these young dependents, attention should be paid to their parents who are often in the age group of 30–39 and 40–49. Furthermore, elasticities of entering probabilities were larger than leaving probabilities in the 0–9, 30–39, and 40–49 age groups, whereas the opposite pattern was shown in the 10–19 and 20–29 age group. These findings inform us that for the 0–9, 30–39, and 40–49 age group, preventing them from entering the CSSA system would be more effective in reducing the number of CSSA recipients; for the 10–19 and 20–29 age group, helping the CSSA recipients leave the system will have a larger impact (see the last column in Table 3). In sum, these results suggest that targeting the age group of 30–49 through the “entering approach” would lead to the greatest reduction in the number of CSSA recipients, as aiming at this group would also effectively prevent those aged 0–9 from entering the CSSA system.

## 5. Discussions

This study investigated the targeting of the social security system in Hong Kong by evaluating the CSSA scheme, which is the fundamental welfare program. We have made use of the Markov chain process to model the dynamics—the inflow and outflow—of the CSSA system. We conducted elasticity analyses to identify the potential target group and the appropriate approaches for different subgroups classified by age. The results have revealed that the largest elasticity was in the 0–9 age group, meaning that targeting children at this age will lead to a largest decrease in the number of CSSA recipients. Currently, child poverty is a serious issue in Hong Kong. In 2017, before government intervention, the poverty rate among children aged below 18 was about 23.1%, much higher than many other developed societies [2,27]. As child poverty often results from parents’ low income and unemployment, policies that support households with children would be desirable. More precisely speaking, the results suggest that targeting those aged 30–49 with children by decreasing the risk of entering CSSA would have the largest impact on the number of CSSA recipients.

Studies on child poverty in Hong Kong have found that family structure is a strong predictor. It was found that children from immigrant families are more likely to live below the poverty line in Hong Kong due to low education of parents, high unemployment rate of mothers, and low payment of parents’ jobs [28]. Moreover, children living with single parents have high risk of poverty; the poverty gap between children with single parents and children with two parents has been widening in recent decades [29]. The ethnic minority families such as Pakistani, Nepalese, and other South Asian countries also have higher child poverty rates, as a result of discrimination in the education system and labor market due to the language barriers faced by their parents [30]. Poverty is found to have a significant impact on children’s psychological well-being—in Hong Kong, children in poverty have reported lower levels of self-esteem and more depressive symptoms, and they are more likely to live in public housing which is often more crowded, having poor hygiene and lack of facilities [31]. Food insecurity, as well as limited educational opportunities and learning resources would have a negative impact on the development of children from poor families, creating intergenerational poverty in Hong Kong [32]. Therefore, it is very important to identify the reasons of falling into the CSSA system and barriers to exit among households with children.

Single parenthood, unemployment, and low-earnings are the major reasons for physically-able adults becoming CSSA recipients. The latest poverty study in 2017 shows that poverty among those who are working was only 4.9% in comparison to 14.7% in the general community [2]. Providing jobs and enhancing job earnings for the age group of 30–49 would effectively reduce the number of CSSA recipients. The Hong Kong government has initiated the Public Transport Fare Subsidy Scheme to enhance people’s ability to look for employment across districts [33]. The government also provides additional support for families—especially single-parent families—to work part-time and look after their children after work. Of course, the employment remuneration conditions should be improved so that working parents can earn enough to support the family without the need of CSSA. The introduction of a minimum wage by the Hong Kong SAR Government in 2011 has helped to narrow the income gap, despite the fact that the minimum wage is still at a very low level of about HKD 37.5 (about USD 4.8) per hour. In many western countries, the minimum wage is substantially higher (e.g., USD 13.3 in Australia, USD 10.3 in the United Kingdom), despite the fact that Hong Kong’s GDP per capita of USD 49,000 actually ranks higher. Thus, it is important to examine the minimum wage structure, especially for those in low-skilled jobs, to improve their quality of life.

Meanwhile, different subgroups call for different approaches. The “entering approach” is not always most desirable for all the subgroups. It is found that for the 10–29 age groups, policies that aim to help people leave CSSA will be more effective than policies that aim to prevent people from entering CSSA. According to the Hong Kong Poverty Situation Report 2017, the unemployment rate among the young people 15–29 is relatively higher than the general population. On the other hand, for those who are employed, young households have the highest proportion of high-skilled workers and the lowest poverty rate [2]. By upgrading working skills of young CSSA recipients, young adults can enhance their employability and secure jobs with better earnings, so that they can support themselves without CSSA. Diversifying the economic and working opportunities is also of great importance in improving the employability of our young people. Most of the economic activities in Hong Kong are in the professional, financial, and service sectors, and limited opportunities are available in other sectors, such as creative industries and local startups. The excessive rental cost in Hong Kong has been found to be a barrier for young people to start their own business. It is important for the government to provide initial support for these young entrepreneurs at the early stage of their career development. Meanwhile, for the 30–59 age groups, policies that aim to prevent people from entering CSSA will be more effective than policies that aim to help people leave CSSA. People of these ages often have other family members to care for, either young children or old parents, and sometimes both. More often, they have jobs but the earnings are not adequate to cover the living expense of the whole family, and thus all members in the household become CSSA recipients. In this case, policies that can increase the family income can prevent them from entering CSSA. We should try to maintain or enhance the employability of these groups of people as a matter of importance. Keeping them in the job is important for poverty alleviation. It will be difficult for the unemployed individuals to reenter to the workforce, especially for those who are not professionals. The bargaining power to aim for better working conditions would also be limited for the low-skilled workers as there are plenty in supply due to the current migration policy, with there being an influx of migrants of 150 per day from mainland China entering into Hong Kong. The wage of the low-skilled worker has been lagging behind the economic growth. Hong Kong still has one of the largest poverty gaps among the high income societies with a Gini coefficient of 0.54 [2]. It is important to improve the wage level, especially for the low-skilled workers in Hong Kong. The minimum hourly wage is HKD 37.5 (USD 4.8), which is one of the lowest in comparison to other OECD (i.e., The Organization for Economic Co-operation and Development) countries. Currently, the Individual-based Work Incentive Transport Subsidy (I-WITS), to some extent, can help middle-aged adults broaden their horizons for better job opportunities across the whole territory [33]. In addition, the Working Family Allowance can provide extra income to households with children. The results have provided insights into the improvement of the CSSA system in terms of accurate targeting. It should be emphasized that the probability of entering and leaving CSSA, as well the elasticities with respect to these probabilities, should be monitored frequently and closely so that a more timely response can be in place to improve the efficiency of CSSA.

In spite of the positives, there are some limitations in this study. First, due to data limitations, we were only able to investigate the targeting groups by age, and did not include the subpopulations by socioeconomic status (e.g., education, employment, occupation). Future research can assess the role of different socioeconomic subgroups in reducing the number of CSSA recipients, with better data access. Apparently, the young graduates from universities have less financial responsibility towards their parents and they can afford to have some gap years before graduation and employment. For the young professionals, once they work, it would be easier for them to leave poverty. Second, we did not take into account gender difference. The number of female CSSA recipients was found to often be larger than male recipients, which is mainly the contribution of women’s overrepresentation in the “single-parent” case under CSSA [34]. For example, in 2017, 87.6% of single-parent recipients were female, most of whom were divorced or separated, in the age of 30–49 [35]. However, due to a lack of gender-specific data, we were unable to examine whether the elasticities were different between men and women, and how big the difference was. Third, we only restricted our analysis to those under age 60 and did not consider the elderly population due to limited information of the elderly people leaving CSSA because of death. Fourth, the Markov chain and elasticity analyses were based on several assumptions (e.g., assumption of the same birth rates between CSSA recipients and non-CSSA people), and consequently they did not fully model the exact dynamics of the CSSA system. Fifth, we did not take into consideration the differential cost in achieving the same unit of the entering and exiting approach. However, this method is still a very useful tool to assess the targeting of the social security system and it can be further elaborated if more information about the CSSA recipients and employment data is available.

## 6. Conclusions

The propose model provides empirical evidence to identify the potential target groups of people that would lead to the largest reduction in the number of the CSSA recipients in the Hong Kong Social Security System. This analytical method has also revealed whether policies would be more effective in preventing people from entering the CSSA system or helping them leave the CSSA scheme. Despite the limitations of the stochastic model, it helps to improve the effectiveness of the social security system in Hong Kong. Poverty and income inequality have become one of the major causes of the recent months of social unrest in Hong Kong in 2019-2020. The Government has been increasing its expenditure on welfare spending. Sometimes, the improvement is still quite limited. Our model can be used as a tool to examine its effectiveness of any poverty program with the aim to reduce the number of CSSA recipients. 

## Figures and Tables

**Table 1:** The estimated age-specific probabilities of leaving and entering CSSA.

Age Group	$p_{t}^{\left(i\right)}\;(\mathrm{Probability}\;\mathrm{of}\;\mathrm{Entering})$	$q_{t}^{\left(i\right)}\;(\mathrm{Probability}\;\mathrm{of}\;\mathrm{Leaving})$
0–9	0.0145	0.1775
10–19	0.0478	0.1356
20–29	0.0015	0.2103
30–39	0.0033	0.1959
40–49	0.0032	0.1379
50–59	0.0028	0.1217

**Table 2:** The estimated probability of first exit and the mean time of staying in the CSSA system.

Age of CSSA Recipients	Probability of First Exit	Mean Time
age 0	0.99998	5.79
age 5	0.99994	6.06
age 10	0.99985	6.77
age 15	0.99968	6.12
age 20	0.99934	4.78
age 25	0.99785	4.85
age 30	0.99300	5.1
age 35	0.97918	5.21
age 40	0.93806	5.72
age 45	0.86992	4.69
age 50	0.72683	3.24
age 55	0.47735	1.31

**Table 3:** The absolute values of rates of change and elasticities of the overall CSSA proportion.

Age Group	Rate of Change		Elasticity		Approach
	$\frac{\Delta X_{t+1}}{\Delta p_{t}^{\left(i\right)}}$	$\frac{\Delta X_{t+1}}{\Delta q_{t}^{\left(i\right)}}$	$\delta_{p_{t}^{\left(i\right)}}$	$\delta_{q_{t}^{\left(i\right)}}$	Leaving vs. Entering
0–9	0.573	0.019	0.221	0.150	Entering
10–19	0.588	0.024	0.126	0.144	Leaving
20–29	0.604	0.012	0.040	0.109	Leaving
30–39	0.656	0.009	0.098	0.079	Entering
40–49	0.624	0.012	0.090	0.075	Entering
50–59	0.316	0.007	0.039	0.038	Entering

## References

[1] United Nations (2016). The Sustainable Development Goals Report 2016.

[2] HKCSD (2018). Hong Kong Poverty Situation Report 2017.

[3] Jones J.F. (1981). The Common Welfare: Hong Kong’s Social Services.

[4] Midgley J. (1984). Social Security Inequality and the Third World.

[5] Legislative Council (2018). The 2018–2019 Budget.

[6] Basic Law (1990). The Basic Law of the Hong Kong Special Administrative Region of the People’s Republic of China.

[7] Lam C. (2007). Speech by the Chief Executive in Delivering “The Chief Executive’s 2017 Policy Address” to the Legislative Council. https://www.policyaddress.gov.hk/2017/eng/speech.html.

[8] Sawada Y. (2004). The social security system in Hong Kong: Establishment and readjustment of the liberal welfare model. Dev. Econ..

[9] Wong H. (2000). The failure of social security in alleviating poverty in Hong Kong. Asia Pac. J. Soc. Work Dev..

[10] Wong H. (2012). Changes in social policy in Hong Kong since 1997: Old wine in new bottles?. Contemporary Hong Kong Government and Politics.

[11] Chiu S., Wong V. (2005). Hong Kong: From familistic to Confucian welfare. East Asian Welfare Regimes in Transition: From Confucianism to Globalization.

[12] Chiu S. (2003). Local Policy in Global Politics: The Limit of Anti-poverty Policy in Hong Kong. J. Soc. Policy Soc. Work.

[13] United Nations (2012). Family-Oriented Policies for Poverty Reduction, Work-Family Balance and Intergenerational Solidarity. https://www.un.org/esa/socdev/family/docs/WorkFamilyBalanceandIntergenerationalSolidarity.pdf.

[14] Lee E.W.Y. (2008). Social mobilization, blame avoidance, and welfare restructuring in Hong Kong. Politics and Government in Hong Kong.

[15] Cassiman S.A. (2008). Resisting the neo-liberal poverty discourse: On constructing deadbeat dads and welfare queens. Sociol. Compass.

[16] Chan C.K. (2011). Hong Kong: Workfare in the world’s freest economy. Int. J. Soc. Welf..

[17] Wiggan J. (2012). Telling stories of 21st century welfare: The UK Coalition government and the neo-liberal discourse of worklessness and dependency. Crit. Soc. Policy.

[18] Ho A.P.-y., Chan K.-t. (2010). The social impact of work-integration social enterprise in Hong Kong. Int. Soc. Work.

[19] Beffy M., Coudin E., Rathelot R. (2014). For Whom are Permanent Jobs off Limits? A Markov-Chain-Based Analysis of Individual Labor Market Dynamics. Ann. Econ. Stat. Ann. ’Écon. Stat..

[20] Bosch M., Maloney W. (2007). Comparative Analysis of Labor Market Dynamics Using Markov Processes: An Application to Informality.

[21] Chen M., Lloyd C.J., Yip P.S. (2018). A new method of identifying target groups for pronatalist policy applied to Australia. PLoS ONE.

[22] Chen M., Yip P.S., Yap M.T. (2018). Identifying the most Influential Groups in Determining Singapore’s Fertility. J. Soc. Policy.

[23] Yip P.S., Chen M. (2016). An elasticity analysis of the effectiveness of pronatalist measures in Taiwan. Asian Popul. Stud..

[24] Gans J., King S., Mankiw N.G. (2011). Principles of Microeconomics.

[25] Vere J.P. (2010). Special Topic Enquiry on Earnings Mobility.

[26] Legislative Council (2015). Social Mobility in Hong Kong. https://www.legco.gov.hk/research-publications/english/1415rb02-social-mobility-in-hong-kong-20150112-e.pdf.

[27] Peng C., Yip P. (2017). How to Break the Cycle of Child Poverty in Hong Kong, Where One in Five Children Are Poor. South China Morning Post. https://www.scmp.com/comment/insight-opinion/article/2095321/how-break-cycle-child-poverty-hong-kong-where-one-five.

[28] Chou K.-L. (2013). Familial effect on child poverty in Hong Kong immigrant families. Soc. Ind. Res..

[29] Cheung K.C.-K. (2015). Child Poverty in Hong Kong Single-Parent Families. Child Ind. Res..

[30] Cheung K.C.-K., Chou K.-L. (2018). Child Poverty Among Hong Kong Ethnic Minorities. Soc. Ind. Res..

[31] Ho K.Y., Li W.H.C., Chan S.S.C. (2015). The effect of poverty and income disparity on the psychological well-being of Hong Kong children. Public Health Nurs..

[32] Qi D., Tang V.M.Y. (2015). Social assistance programs and child poverty alleviation—A comparison between Hong Kong and Mainland China. Asian Soc. Work Policy Rev..

[33] Sha F., Li B., Law Y.W., Yip P.S.F. (2019). Associations between commuting and well-being in the context of a compact city with a well-developed public transport system. J. Trans. Health.

[34] Women’s Commission (2017). Hong Kong Women in Figures 2017. https://www.women.gov.hk/download/research/HK_Women2017_e.pdf.

[35] HKCSD (2018). Statistics on Comprehensive Social Security Assistance Scheme, 2007–2017. https://www.statistics.gov.hk/pub/B71809FB2018XXXXB0100.pdf.

